# Multiple intraductal papillary neoplasms of bile duct diagnosed based on endoscopic ultrasonography and peroral cholangioscopy findings

**DOI:** 10.1007/s12328-024-02000-2

**Published:** 2024-07-06

**Authors:** Masaki Onoe, Nobuhiko Fukuba, Yasuhide Kodama, Akihiko Oka, Kousaku Kawashima, Kotaro Shibagaki, Norihisa Ishimura, Yoshinori Kushiyama, Yasushi Uchida, Toru Furukawa, Shunji Ishihara

**Affiliations:** 1 Department of Gastroenterology and Hepatology, Shimane University School of Medicine, 89-1, Enya-cho, Izumo, Shimane Japan; 2 Division of Endoscopy, Shimane University Hospital, Izumo, Japan; 3 Department of Gastroenterology, Matsue Red Cross Hospital, Izumo, Shimane Japan; 4 Department of Investigative Pathology, Tohoku University Graduate School of Medicine, Sendai, Japan

**Keywords:** Endoscopic ultrasonography, Intraductal papillary neoplasms of bile duct, Peroral cholangioscopy

## Abstract

A man in his 30s was referred to our department for evaluation of bile duct stricture and removal of an intrahepatic bile duct stone. Five years before his presentation, he underwent left hepatectomy for a giant hepatic hemangioma. There were no abnormalities in blood biochemical tests. Magnetic resonance cholangiopancreatography showed one 5 mm oval defect in region B6 and two 8 mm semicircular defects in the hilar bile duct. Endoscopic ultrasound revealed a 3.5 mm hypoechoic focal raised lesion in the hilar bile duct. Oral cholangioscopy revealed his two lesions in the hilar bile duct as white papillary elevations with mucus production. The pathological diagnosis of intraductal papillary neoplasm was determined (low-grade dysplasia, type 1, gastric type). After 1 and a half years, no expansion of the bile duct lesion was observed. Initially, it was thought to be a benign stenosis after liver resection, but based on the results of endoscopic ultrasound, we suspected a tumorous lesion, and we were able to make an accurate diagnosis, including histological type, using transoral cholangioscopy.

## Introduction

Intraductal papillary tumor (IPNB) is a papillary tumor that occurs within the bile duct, and no diagnostic or treatment methods have been established. This case report describes a case of IPNB diagnosed using endoscopic ultrasound and transoral cholangioscopy. Considering the invasiveness and the patient’s medical history, we decided against surgery and opted for careful MRI monitoring. This highlights the evolving role of diagnostic imaging in the management of IPNB and provides insight into treatment strategies.

## Case report

**Fig. 1:**
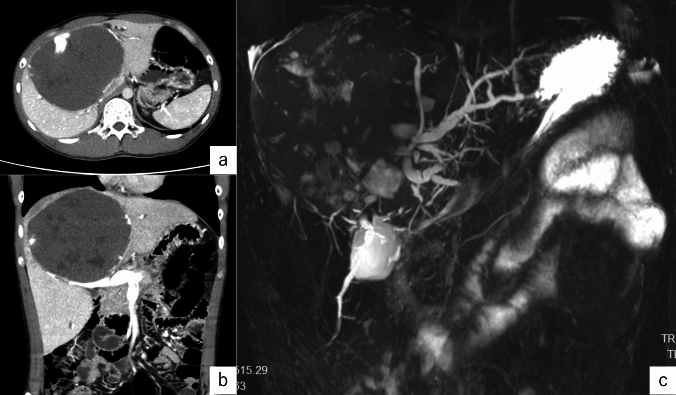
Contrast-enhanced computed tomography **a** horizontal section, **b** coronal section, and **c** MRCP. Five years prior to referral, he diagnosed as a giant hepatic hemangioma at his left medial segment. No findings suggestive of IPNB could be noted at this point due to the displacement by a giant hepatic hemangioma

**Fig. 2:**
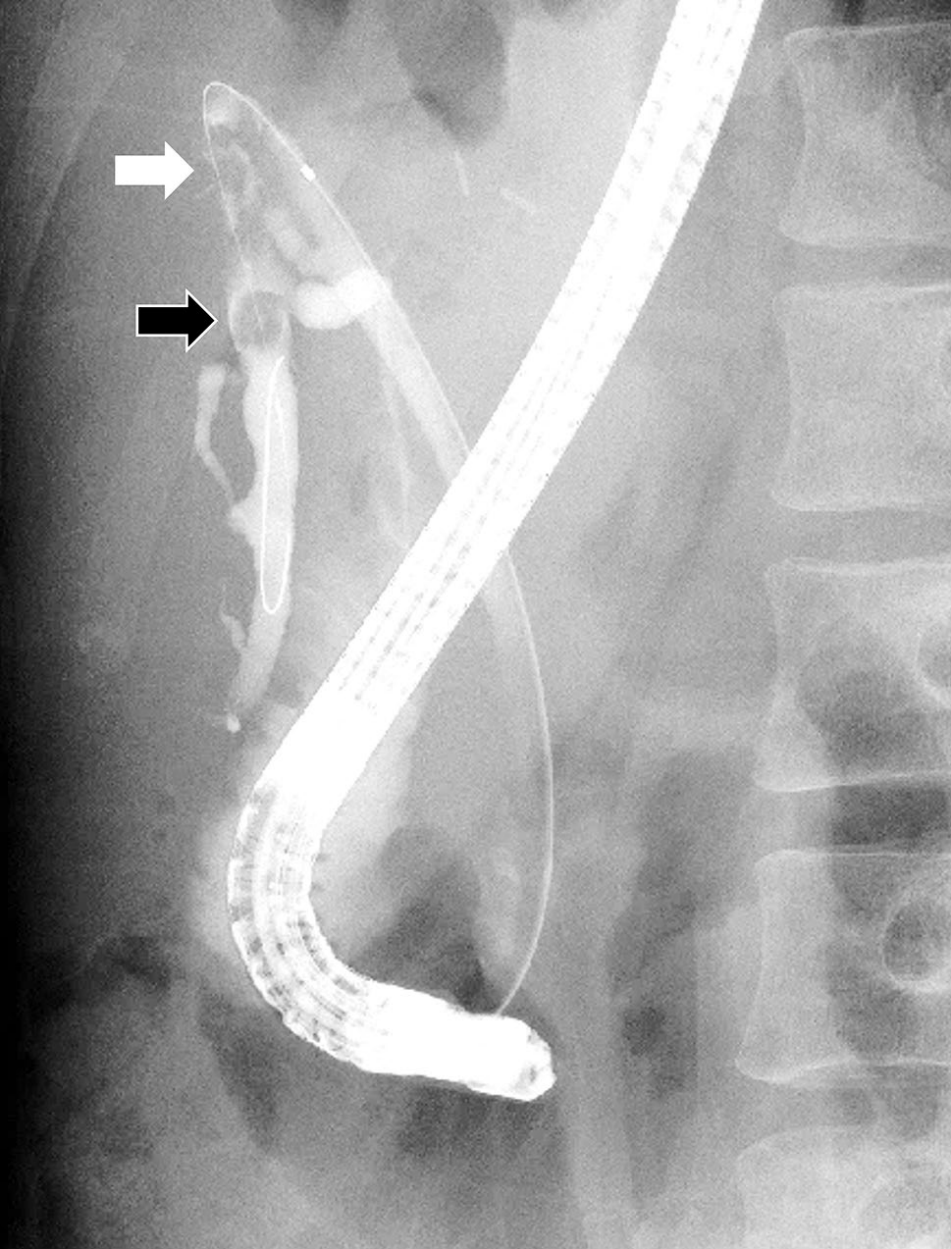
Cholangiography performed at the referring medical institution showed worsening of bile duct stenosis (White arrow) along with intrahepatic stones (Black arrow)

A male in his thirties was referred to our department for evaluation of bile duct stenosis and removal of intrahepatic bile duct stones. The patient had a history of cervical spine deformation, for which he underwent iliac bone transplant surgery in his teens. In his twenties, autoimmune hepatitis was diagnosed, though that improved without steroid treatment. Five years prior to referral, he underwent an expanded left hepatectomy for a giant hepatic hemangioma (Fig. 1). Then one year later, an examination conducted at a primary care facility found multiple bile duct stenosis sites. The diagnosis was benign stenosis due to effects of the hepatectomy, and an endoscopic biliary stent was placed and regularly replaced. Cholangiography at the time of stent replacement showed worsening of bile duct stenosis along with intrahepatic stones and the patient was referred to our hospital (Fig. 2).

Physical examination findings: The patient showed limited forward bending due as a post-cervical surgery complication, clear consciousness, and a flat and soft abdomen without tenderness. A surgical scar was present in the upper abdomen. History of exposure to organic solvents was not noted.

Laboratory test findings: Leukocyte count, 5.30×10^3^/µL; platelet count, 173×10^3^/L; total bilirubin, 1.0 mg/dL; aspartate aminotransferase, 16 U/L; alanine aminotransferase, 13 U/L; alkaline phosphatase, 68 U/L; γ-glutamyl transpeptidase, 23 U/L; amylase, 50 U/L; CRP, 0.05 mg/dL; CEA, 1.6 ng/mL; CA19-9, 10.3 U/mL; IgG4, 152 mg/dL.

**Fig. 3:**
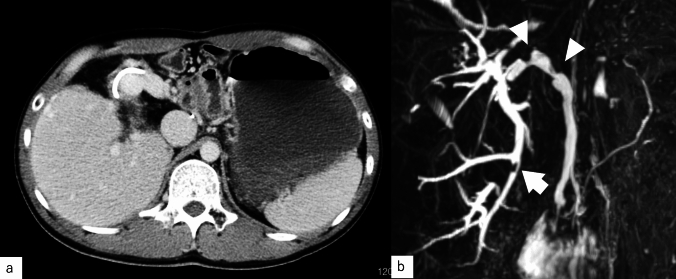
**a** Computed tomography performed after bile duct stent placement at the referring medical institution revealed no residual hemangioma in the posterior segment of the liver, and no masses were detected in the common bile duct or the bile duct in the posterior segment. **b** A single 5 mm oval defect in the B6 region (arrow) and two 8 mm semicircular defects (arrow head) in the hilar bile duct were noted in MRCP imaging findings

**Figure 4:**
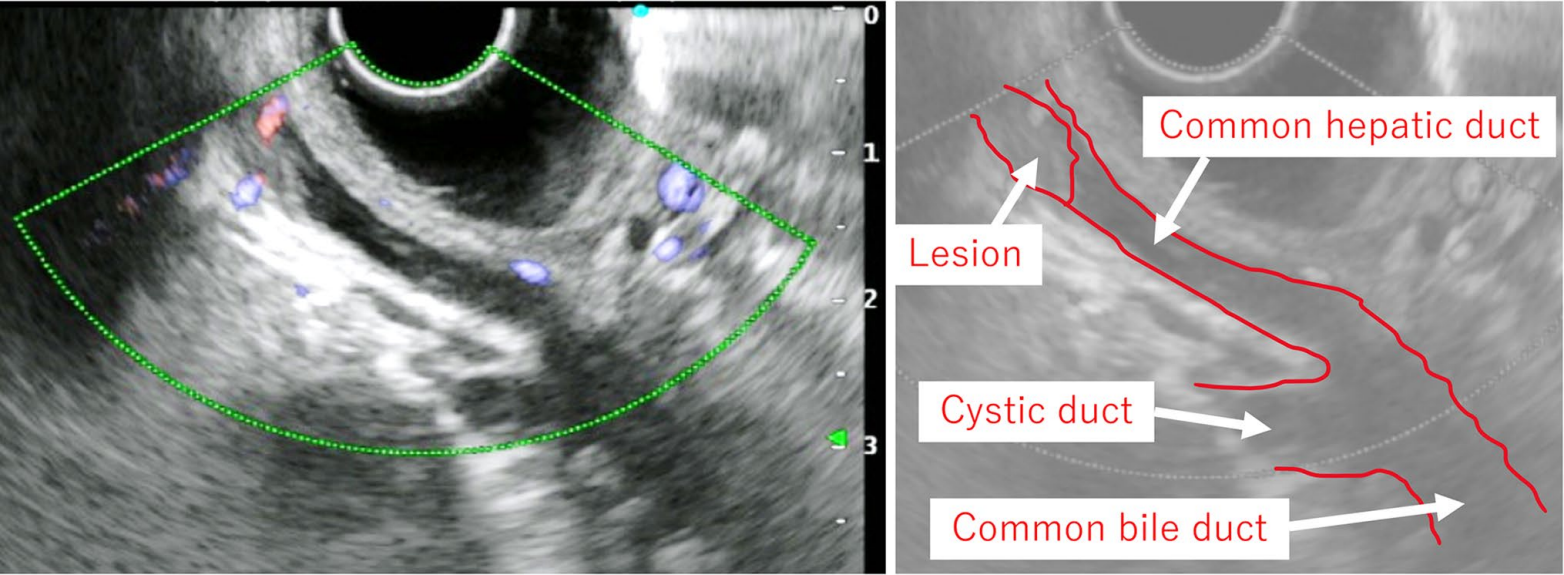
Endoscopic ultrasound viewed from the duodenal bulb revealed a 3.5 mm hypoechoic locally elevated lesion in the hilar bile duct. The figure on the right is a schematic diagram

**Fig. 5:**
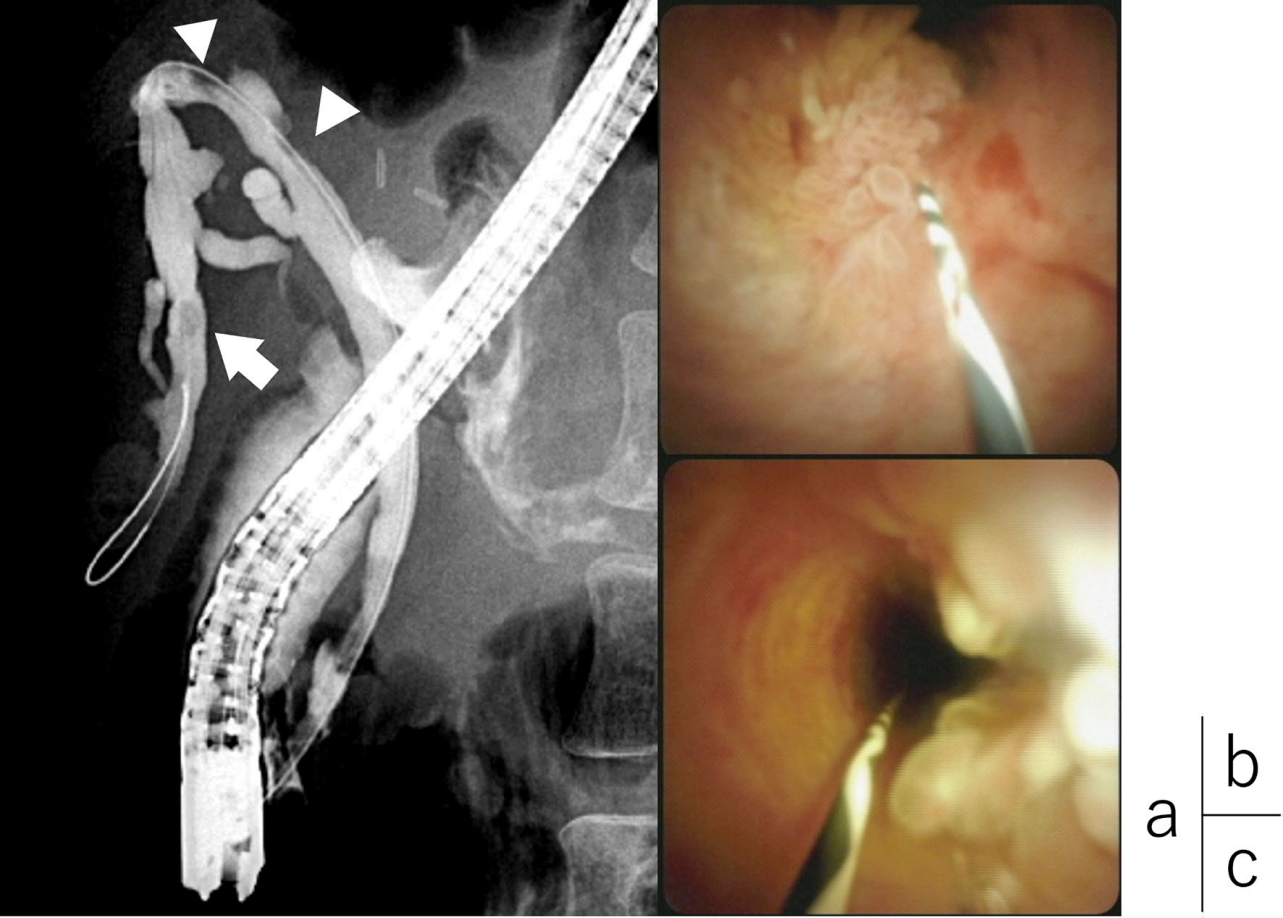
**a** ERCP revealed two areas of bile duct stenosis in the hilar part, similar to MRCP findings. **b**, **c** Using a single-use endoscope (SpyScope DS-2; Boston Scientific, Marlborough, Mass, USA), two lesions in the hilar bile duct were observed as white papillary elevations with mucus production

**Fig. 6:**
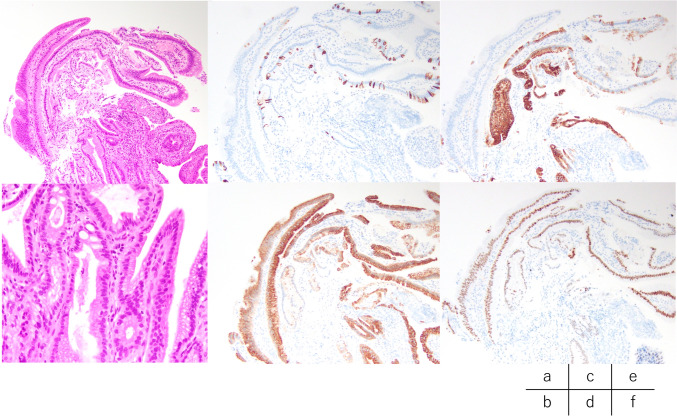
Pathological evaluation using biopsy forceps (SpyBite MAX; Boston Scientific) revealed papillary formation with low-grade dysplastic epithelial proliferation. **a** 40 × and **b** 100 × images of hematoxylin and eosin staining. Immunohistochemical staining showed **c** MUC2 (sparse/metaplastic+), **d** MUC5AC (+), **e** MUC6 (focal+), and **f** CDX2 (focal+). Based on these pathological findings, the diagnosis was IPNB (low-grade dysplasia, Type 1, gastric type)

Imaging findings: Computed tomography revealed no residual hemangioma in the posterior segment of the liver, and no masses were detected in the common bile duct or the bile duct in the posterior segment (Fig. 3a). Magnetic resonance cholangiopancreatography (MRCP) imaging showed a single oval defect 5 mm in size in the B6 region and two 8 mm semicircular defects in the hilar bile duct (Fig. 3b). Endoscopic ultrasound revealed a 3.5 mm hypoechoic localized raised lesion in the hilar bile duct (Fig. 4), which suggested that bile duct stenosis was not a postoperative change as initially suspected but most likely due to a tumor. For confirmation, endoscopic retrograde cholangiopancreatography (ERCP) and oral cholangioscopy were performed. ERCP showed two areas of bile duct stenosis in the hilar part, similar to the MRCP findings (Fig. 5a). For the oral cholangioscopy examination, a single-use cholangioscope (SpyScope DS-2; Boston Scientific, Marlborough, Mass, USA) was used, which revealed two lesions in the hilar bile duct as white papillary elevations with mucus production (Fig. 5b, c). In addition, pathological findings obtained using biopsy forceps (SpyBite MAX; Boston Scientific) showed papillary formation with low-grade dysplastic epithelial proliferation in (Fig. 6a, b), while immunohistochemical staining indicated MUC2 (sparse/metaplastic+), MUC5AC (+), MUC6 (focal+), CDX2 (focal+), and S100P (+) (Fig. 6c–f). At the referring medical institution, the patient was diagnosed with benign stricture after liver resection, but the diagnosis was changed to intraductal papillary neoplasm of bile duct (IPNB, mild dysplasia, type 1, gastric type) based on these pathological findings.

**Fig. 7:**
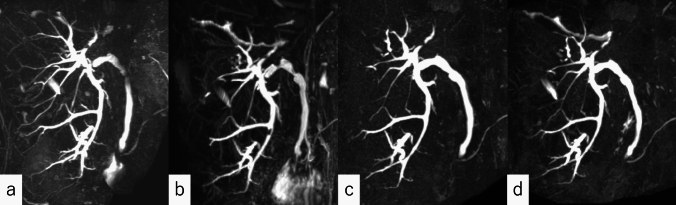
MRCP images are arranged in chronological order. **a** After left hepatic lobectomy, **b** when the patient was referred to our department with a B6 bile duct stone, **c** 6 months after IPNB diagnosis, and **d** 12 months after diagnosis

Following consultation with the hepatobiliary and pancreatic surgery department of our hospital, bile duct resection was considered. However, due to patient history of left hepatectomy, another surgical procedure was considered highly invasive and not acceptable. After a thorough explanation to the patient and obtaining consent, it was decided to remove the intrahepatic bile duct stones with a balloon catheter and then carefully observe the course with follow-up MRI without replacement of the biliary stent. MRI re-examinations performed at 6 and 12 months after stent removal showed no enlargement of the bile duct lesions (Fig. 7). Continued close follow-up is planned.

## Discussion

In 2001, Chen et al. reported the disease concept IPNB as a papillary tumor of the bile duct [1]. Findings for these tumors have a wide spectrum, ranging from benign to malignant, with potential to progress to cholangiocarcinoma [2, 3], and the disease concept has evolved with accumulation of pathological and clinical results. Conditions with similar pathological features but reported under different names (e.g., bile duct adenoma, intraductal papillary tumor) were integrated into a single category as IPNB in the 4th edition of the World Health Organization (WHO) classification of tumors revised in 2010, with detailed descriptions as an independent disease concept added in the 5th edition revised in 2019 [4, 5]. The rate of incidence has been noted to be 10–15% of all biliary tract tumors, with cases in East Asia primarily reported [2, 6–9]. IPNB is more commonly found in middle-aged and older men and often asymptomatic, though may also be related to pain and jaundice [10]. Occurrence has been noted in the intrahepatic and extrahepatic bile ducts, with a tendency to be more common in the left lobe of the liver. In the present case, the initial findings were incidentally noted in results of an imaging examination performed after surgery for a giant hepatic hemangioma in a relatively young patient without symptoms. Notably, skip lesions were revealed, all low-grade dysplasia, Type 1, gastric type. Lesions were only observed in the extrahepatic bile duct using cholangioscopy, though the possibility of skip lesions in the intrahepatic bile duct cannot be denied. Following the diagnosis, a review of liver parenchyma pathology findings from a hepatectomy procedure performed five years prior (not shown) provided no evidence of such intraductal epithelial tumors. Furthermore, although this case had a history of autoimmune hepatitis, reports of autoimmune hepatitis accompanied by papillary lesions in the bile duct indicate that hepatitis inflammation may have contributed to the development of bile duct lesions [11]. However, in this case, the disease had already subsided spontaneously at the time of liver resection, and pathological examination of the liver parenchyma did not reveal any findings suggestive of autoimmune hepatitis. We believe that there is little evidence that autoimmune hepatitis was involved in the pathology of this case.　These intrabilliary lesions did not worsen over the following year and no new lesions were observed in the intrahepatic bile duct, indicating a generally slow-growing tumor that required careful follow-up. Findings obtained in the present case are valuable for speculating the natural history of IPNB.

IPNB is classified into four subtypes, i.e., pancreatobiliary, intestinal, gastric, and oncocytic, based on the morphological characteristics of the cells, which reflects differences in tumor cell differentiation. Clinical behavior varies by subtype, with the gastric type often relatively benign, while the intestinal type and pancreatobiliary type are more likely to progress and shows a tendency to be malignant. Whether there are prognostic differences among the four subtypes is subject to debate, while the presence of multiple subtypes has been reported [12]. The 5th edition of the WHO classification has adopted a new classification proposed by experts from Japan and Korea as an experimental attempt, with IPNB divided into two main types; Type 1, similar to pancreatic intraductal papillary mucinous neoplasm, and Type 2, similar to cholangiocarcinoma [5]. Molecular analysis results show mutations in *KRAS* and *GNAS* enriched in Type 1 IPNB, whereas mutations in *TP53, SMAD4*, and *KMT2C* are enriched in Type 2. These classifications indicate two distinct types of neoplasms specifically associated with clinicopathological features and molecular phenotypes [13]. Furthermore, Type 1 IPNB is characterized by prominent papillary or villous tumor growth often without invasion. Occurrence is more frequently noted in the intrahepatic bile duct, with gastric, intestinal, and oncocytic subtypes common. On the other hand, Type 2 IPNB, associated with higher degrees of dysplasia and invasive potential, poses a higher risk for progression to high-grade malignant tumor. Occurrence is more frequent in the extrahepatic bile duct, with intestinal and pancreatobiliary subtypes common. While Type 1 is often associated with a relatively good prognosis, Type 2 IPNB presents more challenges for treatment and is considered to be a more unfavorable factor for patient prognosis [2]. Therefore, accurate distinction between Types 1 and 2 is critically important for determining appropriate treatment strategies and evaluating prognosis.

The present case showed lesions in the hilar bile duct diagnosed as gastric type, based on mucinous quality and histological imaging, with low-grade dysplasia and considered to be Type 1. Despite age in the thirties and a history of left hepatectomy for a giant hepatic hemangioma, the patient consented to careful follow-up after a thorough explanation regarding the invasiveness of surgical resection. More than one year after diagnosis, no significant changes were observed in MRCP findings. Determination of subtype has a crucial role for deciding treatment strategy to avoid overtreatment. Accumulation of findings regarding long-term prognosis will be helpful.

Diagnosis of IPNB is primarily based on imaging findings, with ultrasound, computed tomography (CT), and MRI useful for assessing tumor location and size, and impact on the bile duct [14]. MRCP in particular has an important role for non-invasive depiction of intrahepatic and extrahepatic bile duct stenoses, especially in cases with multiple IPNBs such as the present. However, its effectiveness is limited for biliary intraepithelial neoplasia (BilIN), which is presented as flat lesions that cause lower levels of less stenosis. Contrast-enhanced CT is effective for evaluating flat lesions that have progressed into the bile duct lumen. Endoscopic ultrasound (EUS), which has a high level of spatial resolution, is effective for revealing the presence and depth of extrahepatic bile duct lesions, as noted in our case, while contrast enhancement with perflubutane also aids in lesion depiction. ERCP is indispensable for confirming diagnosis by providing endoscopic imaging of the bile duct, with cytology or histology results used as needed. Intraductal ultrasonography might also be necessary, but we omitted it to give priority to cholangioscopy. This is because when performing cholangioscopy, we want to reduce contact between the device and the lesion as much as possible. The disposable nature of the Spyscope DS system poses a cost challenge for suspected extrahepatic bile duct stenosis. Nevertheless, for affected cases such as the present, examining the bile duct with convex EUS and suspicious tumorous lesions with use of the Spyscope DS application can be an efficient strategy. On the other hand, for patients without tumor echo findings in the bile duct shown by convex EUS, and who are not presented with jaundice or compromised liver function, a follow-up MRCP examination may be sufficient.

IPNB is considered to be the biliary counterpart of a pancreatic intraductal papillary mucinous neoplasm (IPMN) because of the common developmental stages of the biliary tract and pancreas, and reported similarities [1, 3, 15–17]. However, due to its low incidence and few reports of cases with involved mucin production, consensus on the disease concept has yet to be reached. Differentiation from BilIN and mucinous cystic neoplasm (MCN) of the bile duct can be challenging. IPNB cases show papillary or villous growth within the bile duct, observable not only with gross examination but also with use of imaging techniques. On the other hand, BilIN is presented as flat lesions, with low papillary growth visible under a microscope and distinguished by papillary elevation height. It has been reported that papillary elevation greater than 5 mm suggests IPNB, while 3 mm or less suggests BilIN [18], though a histological examination is required for determining the final diagnosis. The two main precursor lesions leading to cholangiocarcinoma are biliary intraepithelial neoplasia and intraductal papillary neoplasm of the bile duct [19, 20]. MCN of the bile duct, a preinvasive lesion formed by cystic changes, is considered to be a counterpart to pancreatic mucinous cystic neoplasm. Its distinguishing features include the presence of an ovarian stroma beneath the epithelium and lack of communication with the bile ducts. An IPNB communicates with the biliary tract and can be shown by cholangiography, thus making it distinguishable from an MCN. Although the features of an MCN are different from IPNB, patients affected by either have a good prognosis following resection. In the present case, ERCP and MRCP revealed multiple bile duct stenoses, initially considered to be non-tumorous lesions by the referring physician due to postoperative findings. While non-tumorous lesions, such as primary sclerosing and IgG4-related cholangitis, were considered, findings indicating low-echoic raised lesions in the bile duct shown by convex EUS raised suspicion of tumorous lesions, leading to direct observation with a SpyscopeDS and obtaining a biopsy specimen, with the results confirming IPNB diagnosis.

Surgery was previously the standard treatment for IPNB, though in recent years that is generally decided based on tumor malignancy and extent, as well as overall health of the patient [21]. For cases similar to the present diagnosed as IPNB Type 1, which occasionally show low-grade dysplasia with a good prognosis, available treatment strategies range from aggressive surgical intervention to regular follow-up examinations with imaging, with no treatment method established. As for Type 2, aggressive surgical resection is preferable. When deciding on the extent of resection, considering the possibility of skip lesions, noted in the present case, requires caution. For patients considered unsuitable for surgery due to overall condition or underlying diseases, or because of patient choice, regular follow-up with imaging may be selected. Recent studies have investigated the effectiveness of endoscopic radiofrequency ablation for cholangiocarcinoma [22], which may also be applicable to IPNB in the future. Following treatment, regular follow-up is necessary to monitor for recurrence or progression with available modalities, including abdominal ultrasound, contrast-enhanced CT, MRCP, and EUS. Abdominal ultrasound is convenient, though may not depict fine lesions, while contrast-enhanced CT is useful for diagnosing lesion extent, but not suitable as a regular surveillance modality due to exposure and risk of renal function decline. For assessing local progression EUS is useful, though requires sedation and has a higher level of invasiveness, making it unsuitable for regular surveillance. MRCP lacks risks of exposure and renal dysfunction, making it a suitable modality for surveillance, though is not applicable for patients with internal metal or cardiac pacemakers [23]. At the time of writing, the present patient is undergoing follow-up examinations every 6 months with simple abdominal MRI (MRCP, T1WI, T2WI, DWI).

Findings obtained in this case demonstrate the importance of advances in imaging diagnostic technology, which can be used for diagnosis of IPNB and management, as well as regular follow-up examinations. There is no evidence as to whether surgery or observation is sufficient. The natural history of type 1, such as this case, can provide valuable evidence. In the future, IPNB cases like this one should be accumulated and analyzed.

## References

[1] Chen TC, Nakanuma Y, Zen Y, et al. Intraductal papillary neoplasia of the liver associated with hepatolithiasis. Hepatology (Baltimore, Md). 2001;34:651–8. 11584359 10.1053/jhep.2001.28199

[2] Onoe S, Ebata T, Yokoyama Y, et al. A clinicopathological reappraisal of intraductal papillary neoplasm of the bile duct (IPNB): a continuous spectrum with papillary cholangiocarcinoma in 181 curatively resected cases. HPB. 2021;23:1525–32. 33832834 10.1016/j.hpb.2021.03.004

[3] Zaccari P, Cardinale V, Severi C, et al. Common features between neoplastic and preneoplastic lesions of the biliary tract and the pancreas. World J Gastroenterol. 2019;25:4343–59. 31496617 10.3748/wjg.v25.i31.4343 PMC6710182

[4] Bosman FT, International Agency for Research on C. WHO classification of tumours of the digestive system. 4th ed. International Agency for Research on Cancer: World Health Organization classification of tumours; 2010.

[5] Board WHO CoTE, International Agency for Research on C. Digestive system tumours. International Agency for Research on Cancer World Health Organization: The WHO classification of tumours; 2019.

[6] Kubota K, Jang JY, Nakanuma Y, et al. Clinicopathological characteristics of intraductal papillary neoplasm of the bile duct: a Japan Korea collaborative study. J Hepato Biliary Pancreat Sci. 2020;27:581–97. 10.1002/jhbp.785 32511838

[7] Wu X, Li B, Zheng C. Clinicopathologic characteristics and long-term prognosis of intraductal papillary neoplasm of the bile duct: a retrospective study. Eur J Med Res. 2023;28:1. 10.1186/s40001-023-01102-w. 36945047 10.1186/s40001-023-01102-w PMC10029268

[8] Youn JM, Hwang S, Ahn CS, et al. Clinicopathological features and long-term outcomes of intraductal papillary neoplasms of the bile duct of the liver: single-institution experience with 146 patients. J Gastrointest Surg. 2022;26:1394–405. 35141839 10.1007/s11605-022-05268-2

[9] Zen Y, Jang KT, Ahn S, et al. Intraductal papillary neoplasms and mucinous cystic neoplasms of the hepatobiliary system: demographic differences between Asian and Western populations, and comparison with pancreatic counterparts. Histopathology. 2014;65:164–73. 24456415 10.1111/his.12378

[10] Nakanuma Y, Sato Y, Ojima H, et al. Clinicopathological characterization of so-called cholangiocarcinoma with intraductal papillary growth with respect to intraductal papillary neoplasm of bile duct. Int J Clin Exp Pathol. 2014;7(6):3112–22. 25031730 PMC4097227

[11] Saito. Intraductal papillary neoplasm of the bile duct arising from a background of autoimmune hepatitis: a case report. J Med Cases. 2013. 10.4021/jmc1439w.

[12] Nakanuma Y, Jang KT, Fukushima N, et al. A statement by the Japan-Korea expert pathologists for future clinicopathological and molecular analyses toward consensus building of intraductal papillary neoplasm of the bile duct through several opinions at the present stage. J Hepato Biliary Pancreat Sci. 2018;25:181–7. 10.1002/jhbp.532 29272078

[13] Aoki Y, Mizuma M, Hata T, et al. Intraductal papillary neoplasms of the bile duct consist of two distinct types specifically associated with clinicopathological features and molecular phenotypes. J Pathol. 2020;251:38–48. 32100878 10.1002/path.5398

[14] Aslam A, Wasnik AP, Shi J, Sahai V, Mendiratta-Lala M. Intraductal papillary neoplasm of the bile duct (IPNB): CT and MRI appearance with radiology-pathology correlation. Clin Imaging. 2020;66:10–7. 32438236 10.1016/j.clinimag.2020.04.036 PMC7577174

[15] Nakanuma Y. A novel approach to biliary tract pathology based on similarities to pancreatic counterparts: is the biliary tract an incomplete pancreas? Pathol Int. 2010;60:419–29. 20518896 10.1111/j.1440-1827.2010.02543.x

[16] Rocha FG, Lee H, Katabi N, et al. Intraductal papillary neoplasm of the bile duct: a biliary equivalent to intraductal papillary mucinous neoplasm of the pancreas? Hepatology (Baltimore, Md). 2012;56:1352–60. 22504729 10.1002/hep.25786

[17] Zen Y, Fujii T, Itatsu K, et al. Biliary papillary tumors share pathological features with intraductal papillary mucinous neoplasm of the pancreas. Hepatology (Baltimore, Md). 2006;44:1333–43. 17058219 10.1002/hep.21387

[18] Nakanuma Y, Kakuda Y, Uesaka K. Characterization of intraductal papillary neoplasm of the bile duct with respect to the histopathologic similarities to pancreatic intraductal papillary mucinous neoplasm. Gut Liver. 2019;13:617–27. 30982236 10.5009/gnl18476 PMC6860037

[19] Ainechi S, Lee H. Updates on precancerous lesions of the biliary tract: biliary precancerous lesion. Arch Pathol Lab Med. 2016;140:1285–9. 27788047 10.5858/arpa.2015-0396-RS

[20] Lendvai G, Szekerczés T, Illyés I, et al. Cholangiocarcinoma: classification, histopathology and molecular carcinogenesis. Pathol Oncol Res. 2020;26:3–15. 30448973 10.1007/s12253-018-0491-8

[21] Ohtsuka M, Shimizu H, Kato A, et al. Intraductal papillary neoplasms of the bile duct. Int J Hepatol. 2014;2014:1–10. 10.1155/2014/459091 PMC4052179 24949206

[22] Yang J, Wang J, Zhou H, et al. Efficacy and safety of endoscopic radiofrequency ablation for unresectable extrahepatic cholangiocarcinoma: a randomized trial. Endoscopy. 2018;50:751–60. 29342492 10.1055/s-0043-124870

[23] Lee S, Kim MJ, Kim S, et al. Intraductal papillary neoplasm of the bile duct: Assessment of invasive carcinoma and long-term outcomes using MRI. J Hepatol. 2019;70:692–9. 30553839 10.1016/j.jhep.2018.12.005

